# The Two-Photon Absorption Cross-Section Studies of CsPbX_3_ (X = I, Br, Cl) Nanocrystals

**DOI:** 10.3390/nano10061054

**Published:** 2020-05-30

**Authors:** Janusz Szeremeta, Magda A. Antoniak, Dominika Wawrzyńczyk, Marcin Nyk, Marek Samoć

**Affiliations:** 1 Advanced Materials Engineering and Modelling Group, Wrocław University of Science and Technology, 50-370 Wrocław, Poland; magda.antoniak@pwr.edu.pl (M.A.A.); dominika.wawrzynczyk@pwr.edu.pl (D.W.); marcin.nyk@pwr.edu.pl (M.N.); marek.samoc@pwr.edu.pl (M.S.); 2 Saule Technologies, Wrocław Technology Park, Duńska 11, 54-427 Wrocław, Poland

**Keywords:** perovskite nanocrystals, nanophotonics, two-photon absorption, z-scan, quantum dots, nanoparticles

## Abstract

The CsPbX_3_ nanocrystals (NCs) with X = I, Br, Cl, or the mixture of Br:I and Br:Cl in a 1:1 ratio were synthesized and characterized by TEM, DLS, and XRD. Recrystallization of the small luminescent NCs in the metastable cubic phase into bigger orthorhombic nanocrystals was monitored by XRD and identified as the main cause of the nanocolloid coagulation. The recrystallization also leads to a decrease in the photoluminescence quantum yield (QY) of the colloidal solution and shortening of the emission lifetime. The two-photon absorption cross-section σ_2_ values calculated from femtosecond Z-scan measurements were compared with those obtained based on the two-photon excited emission technique. These two techniques were shown to be equivalent with the cross-section values calculated per molar mass of CsPbX_3_ perovskite being in the range of 10–200 GM depending on the halide anions X^−^. The σ_2_ values recalculated for the mole of the NCs were in the range of 10^4^–10^5^ GM, which is in good agreement with values previously reported elsewhere and the σ_2_/M parameter was in the range of 0.01 to 0.33. This study shows the perovskite NCs to be a good nonlinear material with the third-order nonlinear optical susceptibility χ^(3)^ of the NCs on the order of 10^−11^ esu.

## 1. Introduction

In the last few years, perovskite materials such as hybrid organic-inorganic lead halide perovskites (CH_3_NH_3_PbX_3_, X = I, Br, Cl), and in particular their nanosized colloidal particles, gained great interest for application in third-generation solar cells, whose efficiency can exceed 20% [1,2,3]. These newly developed semiconducting materials exhibit strong light absorption, small exciton binding energies, high charge carrier mobility, as well as long charge carrier diffusion length [3]. In addition, perovskites were also found to be efficient light-emitters for application in light-emitting diodes (LED) [4] and lasers [5]. At the beginning, a majority of the research had been focused on the preparation of thin crystalline films but, along with them, extensive studies were undertaken to produce perovskite nanocrystals (NCs) stabilized by organic ligands [6]. In 2015, Protesescou et al. developed a route to obtain all-inorganic cesium lead halide perovskite NCs with a wide color gamut and extremely high, up to 90%, photoluminescence quantum yield (PL QY) [7]. The small quantum confinement effect in those NCs led to a size tunable emission wavelength only in a narrow range, nevertheless, changes in the composition can shift emission from blue for Cl^−^ ions to green for Br^−^, and red for I^−^, while all intermediate colors can be obtained by varying the Cl/Br or Br/I ratio. This can be accomplished during synthesis by changing amounts of the halide precursors, but also in the post-treatment by an exchange of the halides ions already incorporated in the NCs with the ions added to the solution in the form of well soluble salts, or by mixing two types of perovskites [8,9]. A high PL QY, emission color tuning ability, together with a high color purity of perovskite NCs make them attractive candidates for LED applications [10]. In addition to electroluminescence, perovskite NCs show the optically pumped stimulated emission (SE) and amplified spontaneous emission (ASE) with a low threshold and large optical gain both under one- and two-photon excitation [11,12,13,14]. Two-photon pumping has in fact several advantages over one-photon excitation, for instance, a lower risk of photodamage to the sample, the absence of a phase-matching requirement, longer penetration depth in biological media, and higher spatial resolution. However, a good material for two-photon pumped lasers has to show both efficient two-photon absorption and ease of achieving population inversion. Two-photon pumping has become a viable technique for the generation of coherent light by the optical gain in colloidal semiconductor NCs [15].

There have been a few reports on the measurements of the magnitude of the two-photon absorption cross-section for perovskite NCs, which showed results from 10^4^ up to 10^6^ GM, proving that CsPbX_3_ NCs can be very good two-photon absorbers [14,15,16,17,18,19]. While those results sound promising there is a need for more systematic studies of the third-order nonlinear optical properties of such systems, which would use proven techniques and procedures to obtain results that can be compared with those for other materials, including such disparate systems as semiconductors, dyes, polymers and dendrimers, or hybrid systems. The present paper shows results obtained for CsPbX_3_ nanocrystals (NCs) with X = I, Br, Cl, or the mixture of Br:I and Br:Cl in a 1:1 ratio. In our previous publications on multi-photon absorption of colloidal quantum dots (QDs) and other systems we pointed out the importance of extrapolating nonlinear optical parameters obtained from measurements performed on solutions to the pure substance and normalizing those in the proper way [20,21,22,23,24]. In our Z-scan measurements performed on solutions we were using appropriate procedures to subtract the contribution of the solvent and the cuvette walls [25]. Moreover, we discussed the suitability of certain figures of merit, which can be useful in comparison of the two-photon absorber quality between various materials, such as molecular weight or molecular volume scaled strength of the two-photon absorption [26,27].

In principle, multi-photon excitation is one of the most promising approaches to generate coherent emission (lasing) of a shorter wavelength than the pumping laser. The perovskites are promising materials for such applications due to their photostability, low SE threshold, and large optical gain. In addition to the application in photonics for lasing, materials with a strong two-photon absorption are required for two-photon microscopy [28] and light frequency upconversion [29]. The applications of multi-photon excited perovskite NCs can be similar to those proposed for metal chalcogenide QDs, which include the in vitro and in vivo imaging, chemical and bio-detection, photodynamic therapy, photocatalysis, photoelectric conversion, etc. Comprehensive studies of the third-order nonlinear characteristics of this novel group of materials and investigation of ultrafast processes occurring under fs excitation should also give a better understanding of the physics of the perovskite materials that can lead to their further applications in optoelectronics and photonics.

## 2. Materials and Methods 

CsPbX_3_ NCs were synthesized according to the original procedure described by Protesescu et al. [7]. Briefly, the lead halides PbX_2_ (X = I, Br, or Cl) were reacted with a freshly prepared Cs precursor at 170 °C in the inert ambient atmosphere. First, the Cs-oleate was prepared by dissolving 0.16 g cesium carbonate Cs_2_CO_3_ in 6 mL octadecene with the addition of 0.5 mL oleic acid. The solution was degassed at 100 °C under a vacuum for 1 h and stirred at 150 °C in nitrogen atmosphere until cesium salt was dissolved. Next, 0.18 mmol lead halide (I, Br, or Cl) or the mixture of the halides (I:Br or Br:Cl in a 1:1 molar ratio) were dissolved in the mixture of 5 mL 1-octadecene, 0.5 mL oleic acid, and 0.5 mL oleylamine at 100 °C. Additionally, to solubilize PbCl_2_, 0.5 mL trioctylphosphine (TOP) was added to the mixture. The solution was degassed under a vacuum for 1 h and heated to the reaction temperature under the nitrogen atmosphere in a Schlenk line. An amount of 0.4 mL freshly prepared Cs precursor solution was injected. Immediately after the injection the change of the mixture color was observed and the reaction was quenched by immersing the flask in an ice bath. Finally, the obtained nanoparticles were centrifuged. The supernatant was discarded and the sediment was redispersed in anhydrous toluene inside a glovebox. Samples were stored there and the aliquots were taken in sealed cuvettes for further studies. All chemicals were purchased from Sigma-Aldrich (St. Louis, MO, USA). 

The morphology of the synthesized CsPbX_3_ NCs was investigated with a Titan Cubed G2 60–300 high resolution transmission electron microscope (HR-TEM) (FEI, Hillsboro, OR, USA) equipped with an ChemiSTEM energy-dispersive X-ray (EDX) spectrometer (FEI, Hillsboro, OR, USA). The NCs size was measured by dynamic light scattering (DLS) using Zetasizer Nano ZS (Malvern Panalytical, Malvern, UK). Cu K-α X-ray diffraction (XRD) patterns were obtained with a MiniFlex 600 (Rigaku Corp., Tokyo, Japan), Absorption and emission spectra, emission lifetime, and PL QY were measured with an FS5 spectrofluorometer (Edinburgh Instruments Ltd., Livingston, UK) for the diluted colloidal solution of the NCs in 1 cm quartz cuvette. PL QY was measured with a dedicated integrated sphere. Fluorescence decays were measured by a time-correlated single-photon counting (TCSPC) technique using a 405 nm, ps pulse laser diode as a light source. 

To determine the nonlinear optical properties (NLO) of the perovskite NCs, two different techniques were used: Z-scan and two-photon excited luminescence (TPEL). Both experiments being performed with a femtosecond laser system, operated at the repetition rate of 1 kHz, consisting of a Integra-C Ti:sapphire regenerative amplifier (Quantronix Corp., East Setauket, NY, USA) coupled with Palitra-FS optical parametric amplifier (Quantronix Corp., East Setauket, NY, USA), which allowed for tuning of the wavelength. In the first experiment, the nonlinear refractive index (n_2_) and the nonlinear absorption coefficient (α_2_) were measured by the closed-aperture (CA) and open-aperture (OA) Z-scan techniques introduced by Sheik-Bahae et al. [30]. This allowed the calculation of the effective nonlinear absorption cross-section (σ_2_). The detailed description of the experimental setup can be found in our previous papers [31]. The analyzed Z-scans were taken in the 750–925 nm range. The samples of colloidal solutions were prepared in a 1 mm thick glass cuvette and the concentration of the perovskite NPs in anhydrous toluene was estimated by gravimetry to be ca. 0.2% varying from sample to sample. In order to exclude the contributions of solvent and cuvette cell to the values of NLO parameters, we have used a calculation method, in which the real and imaginary parts of the cubic hyperpolarizability of the species present in the solution are derived from the differences between the Z-scans traces obtained for the cell filled with a pure solvent and the cell with the solution of the investigated perovskite NCs. In the second experiment, the TPEL efficiencies of the investigated samples were compared to those of reference dyes, the relations given by Makarov et al. [32] together with the listed there σ_2_ values for the dyes selected as the references were employed. Due to the required overlap of the NCs and organic dyes emission spectra, we have used fluorescein, rhodamine B, and perylene as references. The spectra of the two-photon excited emission were collected by an Ocean Optics QE Pro-FL fiber optic spectrograph. The range of the laser wavelengths used for the excitation was 575–925 nm. The concentrations of both the organic dyes and the NPs were chosen to be low enough to eliminate internal filter effects and errors arising from uneven distribution of the excited species in the detected volume. We described this method in more detail in our previous studies of the NLO properties of the colloidal quantum dots (QDs) [20].

## 3. Results and Discussion

CsPbX_3_ NCs with five various contents of halides were synthesized: CsPbI_3_, CsPbBr_1.5_I_1.5_, CsPbBr_3_, CsPbBr_1.5_Cl_1.5_, and CsPbCl_3_. No attempt of synthesizing CsPbCl_1.5_I_1.5_ was undertaken since as stated by Akkerman et al. [9], the difference between the size of the iodine and chlorine anions is too big and mixing of Cl^−^ and I^−^ involve a structural stress on the perovskite lattice that cannot be tolerated in the NCs. We did not determine the actual content of the halide in the NCs, however, the theoretical ratio of the Br:I and Br:Cl is 1:1 (x = 1.5). This is the ratio of lead halides used for the synthesis and we assume that the lead halides were completely reacted because the Cs precursor was used in double excess. 

**Figure 1:**
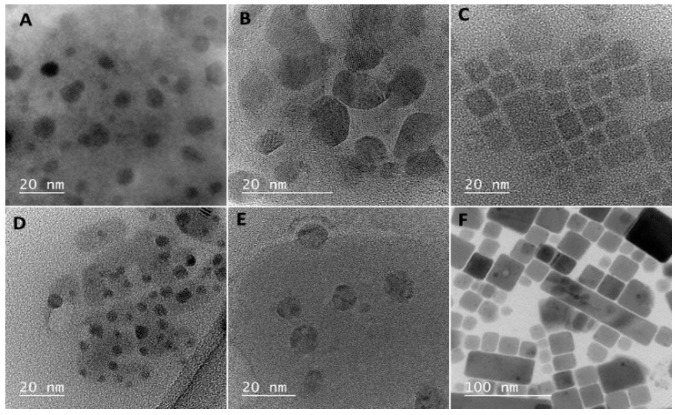
TEM images showing the (**A**) CsPbI_3_, (**B**) CsPbBr_1.5_I_1.5_, (**C**) CsPbBr_3_, (**D**) CsPbBr_1.5_Cl_1.5_, (**E**) CsPbCl_3_, and (**F**) CsPbBr_3_ precipitate.

The morphology of nanocrystals was investigated by TEM imaging. The pictures revealed that NCs are irregular in shape and their aggregates are surrounded by an organic material (Figure 1), most likely the oleylamine or oleic acid used during the synthesis. HRTEM pictures show a well-defined crystalline lattice in the NCs. The EDS spectra of the nanocrystals can be found in Figure S1 in Supplementary Materials. The size of the NCs was measured by DLS and the average size of the NCs was in the range of 16–22 nm (see Figure S2 in Supplementary Materials) and the results are summarized in Table 1. 

**Figure 2:**
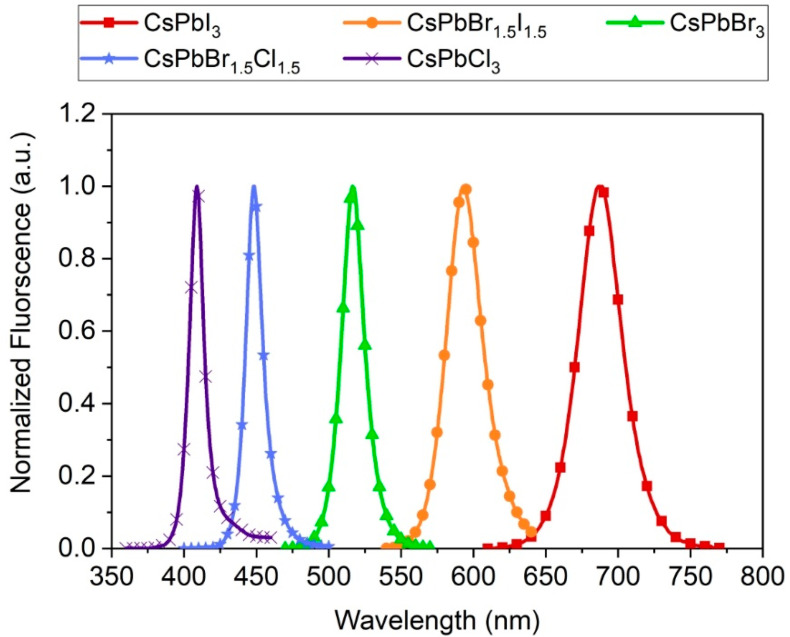
Normalized emission spectra of the CsPbX_3_ NCs with various halide contents upon excitation with the Xe lamp at 350 nm.

**Figure 3:**
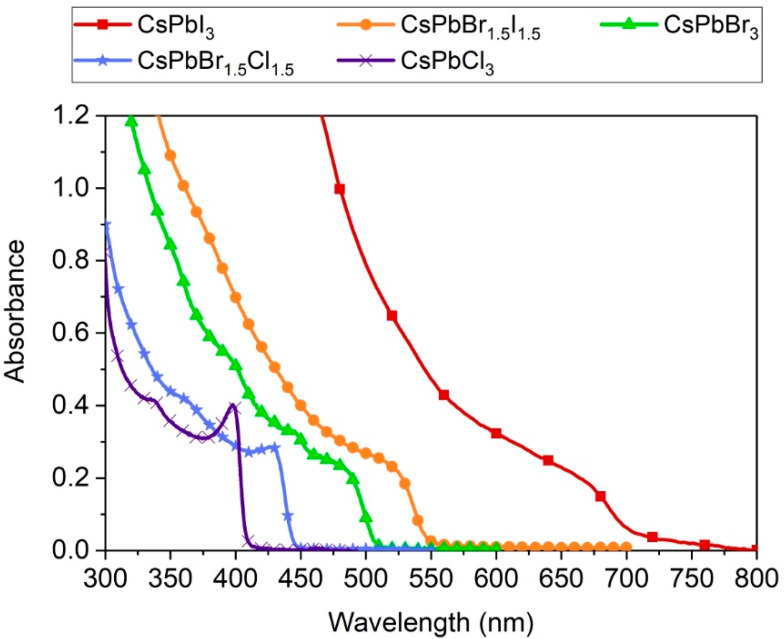
Absorption spectra of the CsPbX_3_ NCs with various halide contents.

**Figure 4:**
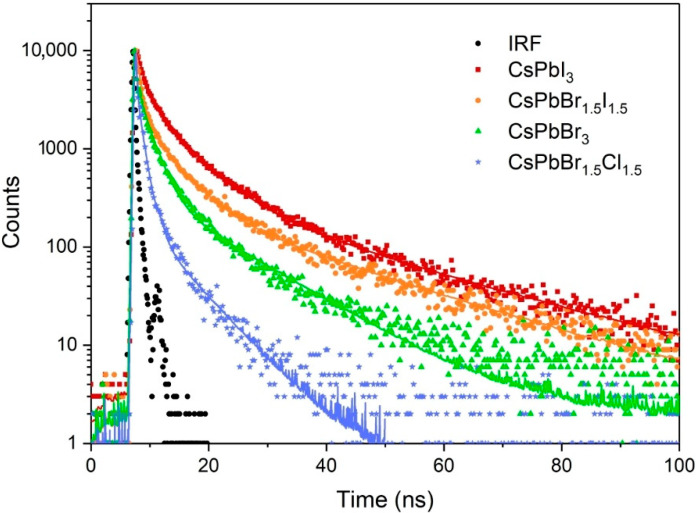
Fluorescence decay measured with the time-correlated single-photon counting (TCSPC) technique with the use of the 405 nm ps laser diode of the CsPbX_3_ NCs with various halide contents.

The studied CsPbX_3_ perovskite NCs do not show the quantum confinement effect such as for e.g., the semiconducting quantum dots. Instead, their spectroscopic properties strongly depend on the halide that constitute the crystals and mixing of the anions shifts the emission spectra [7,8]. Starting from Cl through the mixture of Cl and Br to pristine Br and through the mixture of Br and I to pristine I the whole visible range can be covered. The maxima of emission of synthesized nanocrystals were at 409, 448, 516, 593, and 687 nm and the emission spectra are presented on the graph in Figure 2. These findings are in agreement with the data presented by other researchers on the perovskite nanocrystals with the same reaction stoichiometry. Narrowing of the bandgap of the perovskite and manifests analogously in the shift of the absorption spectra (Figure 3). The obtained QY of the perovskite nanocrystals were rather low and did not exceed 10% (Table 1), the best reported values being as high as 50–90% [7]. This is consistent with the results of fitting of measured fluorescence decays (Table 1). The decays presented in Figure 4 could be best fitted with three-exponential curves. As reported in previous studies [7,18], the emission lifetimes of the NCs are shorter the more emission is shifted towards the blue and range from 6.5 to 24 ns. For our synthesized NCs two shorter decay components appear for each decay, the first below 1 ns and the second ranging from 1–6 ns. All this leads to the conclusion that the emission of the obtained NCs was quenched. The culprits can be both the defects in the NCs lattice in the bulk, or surface defects and the impurities and synthesis precursors leftovers in the colloidal solutions. The repetition of the purification step after the synthesis did not increase the lifetime of the NCs, however, it caused problems with NCs redispersion and accelerated sedimentation. 

**Figure 5:**
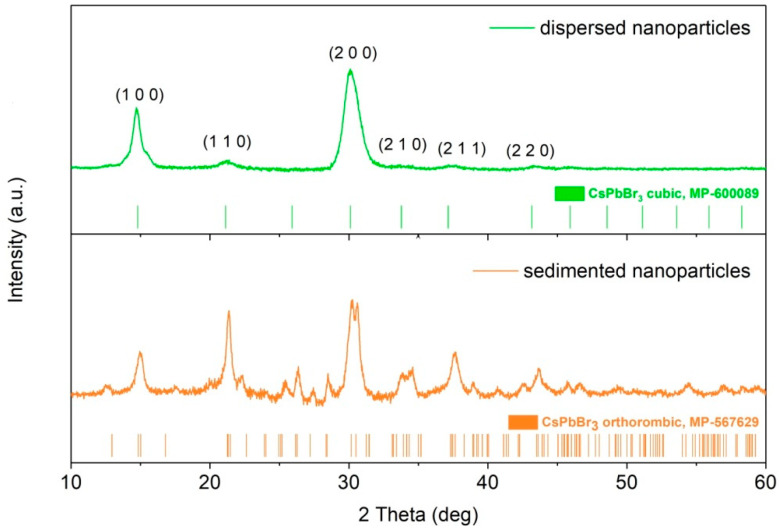
XRD pattern of the CsPbBr_3_ NPs taken from the supernatant and sedimented fraction of the NCs.

We compared the TEM images obtained for the sedimented and dispersed fraction of the CsPbBr_3_ NCs taken from the same solution. Figure 1F presents an image of the larger sedimented particles with cuboid shapes with lengths from 18 to 142 nm. Surprisingly, they do not resemble the aggregates of the smaller particles but rather the individual, single crystals. XRD analysis revealed that well dispersed smaller NCs have a pure cubic crystallographic phase with three main characteristic planes (100) at 14.7°, (110) at 21.4°, and (200) at 30.1° [33,34]. A fraction of the large NCs has the orthorhombic phase, which manifests itself in the splitting of the main peak at 30.1° and the appearance of additional peaks in Figure 5. This shows that precipitation is not the result of aggregation but rather recrystallization of smaller cubic particles, which at room temperature are in the metastable state, into larger orthorhombic particles. It is known that the optically inactive orthorhombic phase is the stable state for bulk lead perovskites at room temperature and transformation from the high temperature cubic phase can be induced by humidity [35,36]. This finding may well explain the low emission efficiency that we observe in the colloidal solution of the mixture of both types of particles. Although we kept the synthesized NCs in the anhydrous solvent in the glovebox and the optical measurements were performed in sealed cells, some exposure of the nanoparticles to the humid ambient air during the transfer of samples and while performing some of the optical measurements could have occurred. Although perovskite materials are promising, the stability of the perovskite nanostructures is a major problem, which can limit their applications and much work has already been done concerning this issue [37,38]. 

Aggregation and sedimentation of the NCs was also an important issue during the NLO measurements. Unfortunately, the samples lacked the long term stability and signs of the degradation were observed under high power laser light illumination. This can be caused by insufficient thermal and photostability of the nanocrystals in ambient air during the measurements. The thermal stability can also be an obstacle during the laser illumination especially when using high power pulse lasers. This is why we prefer to use in our measurements a low repetition rate femtosecond laser instead of e.g., a nanosecond source, because ultra-short pulses cause less heating of the material and allow one to avoid nonlinear optical effects having a thermal origin as well as degradation of the material. Studies can be found showing good thermal and photostability of the CsPbX_3_ (X = Cl, Br, I) perovskite nanocrystals [39,40]. A recent study of thermal stability by Zhang et al. [41] using the in situ TEM imaging shows that all-inorganic halide perovskites have a superb stability up to 690 K. High temperature also induces the transfer into an optically active cubic phase, which is metastable at room temperature but it is possible that even higher temperatures can be reached in the focus of the laser beam and, at the extreme, this can even lead to nanoparticles melting. Although care was taken not to reach such conditions, the need to obtain measurable NLO effects had set a relatively narrow range of intensities where such a compromise could be established. This has led to a relatively large scatter of the results and, therefore, we cannot discuss here the shapes of the full α_2_ vs. wavelength spectra as we did for other NCs, in particular semiconductor quantum dots, and we limit the discussion to just the maximum values obtained for the NCs with both the Z-scan and TPEL techniques. We note that the NCs of CsPbBr_3_ were the most stable during the optical measurements. 

To compare the strength of two-photon absorption of various species usually the macroscopic two-photon absorption coefficient α_2_ values are converted to those of microscopic two-photon absorption cross-section σ_2_. In the case of organic molecules such calculations include the molar mass of the chemical based on its chemical structure, but it is not that obvious in the case of other species, e.g., polymers whether the molar mass of the monomer or average molar mass of the whole chain should be reported [31]. Similarly, in the case of nanoparticles, the molar mass based on the chemical formula can be used for the calculation or the whole particle can be treated as a single entity and average mass of the mole of nanoparticles can be used instead. Fortunately, both approaches lead to the same value of the normalized cross-section σ_2_/M, which is an important merit factor for some applications of nonlinear absorption [20,21,22,23,24]. Here, we decided to use the chemical formula of the perovskite to compare the results obtained with Z-scan with corresponding ones calculated based on two-photon excited emission. We also recalculated the Z-scan results per mole of nanoparticles to compare our results with other reports as well as the parameter of two-photon absorption cross-section divided by molar mass σ_2_/M for comparison with other types of NLO materials. The maximum σ_2_ calculated from the spectra of the two-photon induced fluorescence for measured perovskite NCs was in range of 23–114 GM while for Z-scan it was 10–170 GM. The obtained values are compared in Table 2. This gives values of σ_2_/M in the range 0.016 to 0.33, which is comparable with previously reported values for the semiconducting quantum dot [42]. The representative Z-scan traces of the CA and OA scans and spectra of two-photon induced emission are shown in the Supporting Information (Figures S3 and S4; respectively). It is not straightforward to calculate the molar mass of the synthesized CsPbX_3_ NCs, because of their dispersion of the size, and differences of the crystal density between cubic and orthorhombic phase, as well as mixed content of the halides. However, we evaluated the mass of the mole of nanocrystals MNPs based on the crystallographic parameters of the crystal cell for the cubic structure of perovskites [33,43,44,45,46] and the average size of NCs that was estimated. Recalculated values of the cross-section for two-photon absorption referred to a whole nanoparticle were on the order of 10^4^–10^5^ GM. These are in very good agreement with the report of σ_2_ for CsPbX_3_ NCs with a mixed halide content by Pramanik et al. [19]. To the best of our knowledge most other studies have focused on CsPbBr_3_ NPs as the most stable ones and the presented values are ~10^5^ GM [15,16,17,18]. Xu et al. reported even an order of magnitude greater value of ~2.7 × 10^6^ GM [14]. It should be noted that Z-scan provides information not only about two-photon absorption but also on the nonlinear refraction, which leads to effects such as self-phase modulation, self-focusing, or self-defocusing of light. The strength of these effects depends on the value of the imaginary and real part of the nonlinear refractive index, which relates to the nonlinear absorption and refraction, respectively. It should be noted that the refractive nonlinearity of the nanocrystals is obtained from the differences between the nonlinear phase shift obtained for a cuvette with the solvent and that for a cuvette with the nanoparticles suspension, therefore, its values usually carry relatively big errors, but they could be estimated in many cases and the relevant nonlinearity parameters could be extrapolated to values for a hypothetical material composed of neat nanoparticles. The nonlinear refractive index obtained in such a way was mostly positive for all the investigated nanocrystals and its maximum values were in the range of 10^−13^ cm^2^/W but the largest value of the nonlinear refractive index calculated for the CsPbBr_3_ NCs was as high as 1.35 × 10^−12^ cm^2^/W (Table 3). It can be noted that this value is almost four orders of magnitude higher than that of silica glass (n_2_ = 2–3 × 10^−16^ cm^2^/W), which was used as the standard in our measurements. The nonlinear refraction ability of a material can be presented as the macroscopic nonlinear refraction index n_2_ or, following Balu et al. [47] and our previous publication [20], as the microscopic nonlinear refraction cross-section σ_R_. The latter parameter is analogous to the σ_2_ and is also given in cm^4^ s or “refractive Göppert–Mayer” (RGM = 10^−50^ cm^4^ s). This allows one to easily compare the refractive and absorptive parts of the third-order nonlinear optical susceptibility χ^(3)^ of the material (σ_R_ is related to the real part of χ^(3)^ while σ_R_ is related to its imaginary part), and the ratio σ_R_/σ_2_ is a useful, unitless figure of merit, relevant, e.g., for optical switching applications. For our synthesized CsPbX_3_ NCs this value is in the range of 1–100.

It should be noted that one often finds in the literature that NLO parameters of some nanosystems are quoted as bulk values (n_2_, α_2_, or χ^(3)^), but describing the properties of a system containing a matrix or solvent and nanoparticles at a certain concentration. To compare such results with those for other systems or coming from other laboratories one needs to either extrapolate that to the bulk materials containing nanoparticles alone or refer to the microscopic properties.

The nonlinear refractive and absorptive properties can be also presented as real and imaginary parts of the complex third-order nonlinear optical susceptibility χ^(3)^ of the material. Under some circumstances it is also the modulus of the complex optical susceptibility that may be important (e.g., it is relevant in the degenerate four-wave mixing phenomenon). We estimate here the value of the real and imaginary parts of χ^(3)^ as well as the modulus of the susceptibility for the measured perovskite NCs to be in the range of 10^−12^–10^−11^ esu. It is seen from the values in Table 3 that the high values of the modulus originate in the strong positive nonlinear refraction rather than the nonlinear absorption. These values are comparable with inorganic semiconductor quantum dots and good organic third-order NLO materials [27,42].

## 4. Conclusions

CsPbI_3_, CsPbBr_1.5_I_1.5_, CsPbBr_3_, CsPbBr_1.5_Cl_1.5_, and CsPbCl_3_ were synthesized and dispersed in anhydrous toluene. Based on the XRD studies their colloidal instability was proven to be due to change in the crystallographic phase from cubic to orthorhombic, which is known to be the perovskite stable phase at room temperature. The measured values of the QY of the colloidal solutions were 0.7–10% with the luminescence lifetimes in the range of 6–24 ns with the presence of two fast quenching process components: one in the time range of 1–6 ns and a second below 1 ns. Both the Z-scan and two-photon excited emission were found to be useful for the determination of σ_2_ and the obtained values are in the range of 10–170 GM depending on the halide ions. On the other hand, the σ_2_ values recalculated for the mole of the NCs were in the range of 10^4^–10^5^ GM, which is in good agreement with previously reported values while the σ_2_/M parameter in the range of 0.01 to 0.33 is similar to the values for semiconductor quantum dots. These values combined with the macroscopic third-order nonlinear optical susceptibility χ^(3)^ of the NCs on the order of 10^−11^ esu demonstrate that the CsPbX_3_ perovskite NCs are a good nonlinear material comparable with the organic two-photon absorbers and other nanomaterials such as semiconducting quantum dots. However, the main challenge enabling their wider application is to improve the phase stability of cubic nanocrystals.

## Figures and Tables

**Table 1:** Comparison of the emissive properties of the CsPbX_3_ nanocrytals (NCs) with various halide contents.

Perovskite Formula	Diameter from DLS (nm)	Max. Emission λ (nm)	Quantum Yield (%)	Emission Lifetime (ns)		
				$\tau_{1}$	$\tau_{2}$	$\tau_{3}$
**CsPbI_3_**	16.3 ± 2.8	687	7.3	0.96 ± 0.01	5.65 ± 0.04	23.6 ± 0.2
**CsPbBr_1.5_I_1.5_**	19.8 ± 3.3	593	6.2	0.55 ± 0.01	4.55 ± 0.04	22.1 ± 0.15
**CsPbBr_3_**	19.6 ± 3.0	516	8.1	0.03 ± 0.01	2.03 ± 0.01	12.2 ± 0.08
**CsPbBr_1.5_Cl_1.5_**	16.1 ± 3.1	448	4.0	0.01 ± 0.01	1.03 ± 0.01	6.58 ± 0.08
**CsPbCl_3_**	21.7 ± 2.6	409	0.7	NA	NA	NA

**Table 2:** Nonlinear optical cross-section of the CsPbX_3_ perovskite NCs.

Perovskite Formula	$\sigma_{2}^{Z-scan}$ (GM)	$\sigma_{2}^{TPEL}$ (GM)	$M_{CsPbX_{3}}$ (g/mol )	$\sigma_{2}^{Z-scan}/M$ (GM×mol/g)	$M_{NCs}$ (g/mol )	$\sigma_{2}^{Z-scan}\left(M_{NCs}\right)$ $\left(10^{4}\;GM\right)$
CsPbI_3_	54 ± 7	NA	720.8	0.075 ± 0.012	5.7 × 10^5^	5.5 ± 0.6
CsPbBr_1.5_I_1.5_	10 ± 2	114 ± 32	610.0	0.016 ± 0.002	8.3 × 10^5^	1.4 ± 0.2
CsPbBr_3_	97 ± 9	67 ± 16	579.8	0.17 ± 0.02	8.4 × 10^5^	14.1 ± 1.4
CsPbBr_1.5_Cl_1.5_	170 ± 9	23 ± 4	512.6	0.33 ± 0.02	7.9 × 10^5^	26.5 ± 1.4
CsPbCl_3_	10 ± 1	NA	446.5	0.022 ± 0.002	8.8 × 10^5^	2.0 ± 0.2

$\sigma_{2}^{\mathrm{TPEL}}$—Two-photon absorption cross-section calculated from Z-scan; $\sigma_{2}^{Z-\mathrm{scan}}$—Two-photon absorption cross-section calculated from two-photon excited luminescence; $M_{{\mathrm{CsPbX}}_{3}}$—Molar mass based on the chemical formula of the perovskite; $M_{NCs}$—Molar mass of the perovskite nanocrystals.

**Table 3:** Nonlinear optical refractive properties of the CsPbX_3_ perovskite NCs.

Perovskite Formula	$\sigma_{R}$ $\left(10^{4}\;RGM\right)$	$Max.\;n_{2}$ $\left(10^{-13}\;cm^{2}/W\right)$	$\left|\chi^{\left(3\right)}\right|$ $\left(10^{-12}esu\right)$	$\sigma_{R}/\sigma_{2}\;$ $=Re\left(\chi^{\left(3\right)}\right)/Im\left(\chi^{\left(3\right)}\right)$
CsPbI_3_	29 ± 6	1.9 ± 0.8	1.0 ± 0.05	5.3
CsPbBr_1.5_I_1.5_	92 ± 16	3.6 ± 0.9	2.1 ± 0.06	65.7
CsPbBr_3_	347 ± 42	13.5 ± 1.6	7.8 ± 0.1	24.6
CsPbBr_1.5_Cl_1.5_	71 ± 38	2.6 ± 0.4	1.5 ± 0.08	2.7
CsPbCl_3_	120 ± 23	3.3 ± 0.5	1.9 ± 0.05	60

## Supplementary Materials

The following are available online at https://www.mdpi.com/2079-4991/10/6/1054/s1. Figure S1. DLS measurements taken for (a) CsPbBr_3_ sedimented particles, (b) CsPbBr_3_ well dispersed particles, (c) CsPbBr_1.5_Cl_1.5_, (d) CsPbBr_1.5_I_1.5_, (e) CsPbI_3_, and (f) CsPbCl_3_; Figure S2. EDX spectra taken during the TEM imaging of (a) CsPbI_3_, (b) CsPbBr_1.5_I_1.5_, (c) CsPbBr_3_, (d) CsPbBr_1.5_Cl_1.5_, and (e) CsPbCl_3_; Description of the Z-scan and TPA results analysis and equations used; Figure S3. Closed aperture (CA) Z-scan traces with theoretical fits recorded using an 850 nm fs laser beam for (a) silica glass, (b) toluene, (c) CsPbBr_3_ NCs, and (d) simultaneously recorded open aperture (OA) Z-scan CsPbBr_3_ NCs; Figure S4. Spectra of the two-photon excited emission of the (a) CsPbBr_3_ NCs and (b) fluorescein excited with the tunable fs laser.
