# Supplementary material for: The Two-Photon Absorption Cross-Section Studies of CsPbX3 (X = I, Br, Cl) Nanocrystals
Source: Nanomaterials (Basel). 2020 May 30;10(6):1054. doi: 10.3390/nano10061054 (PMC7352535; doi:10.3390/nano10061054)
Supplement: Supplementary file 1 [file nanomaterials-10-01054-s001.pdf]

Supplementary Materials

The two-photon absorption cross-section studies of $CsPbX_3$ (X = I, Br, Cl) nanocrystals

Janusz Szeremeta [1,2,*], Magda A. Antoniak [1], Dominika Wawrzyńczyk [1], Marcin Nyk [1] and Marek Samoć [1]

1. Advanced Materials Engineering and Modelling Group, Wrocław University of Science and Technology, Wybrzeże Wyspiańskiego 27, 50-370 Wrocław, Poland;
2. Saule Technologies, Wrocław Technology Park, Duńska 11, 54-427 Wrocław, Poland;

* Correspondence: janusz.szeremeta@sauletech.com;

1. Morphology of the nanoparticles

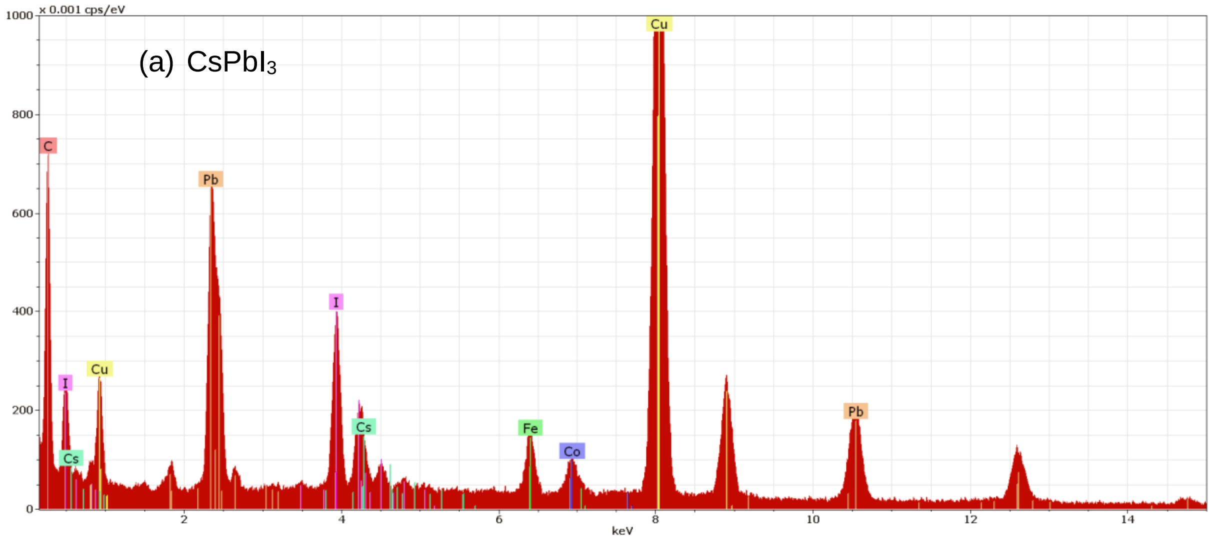

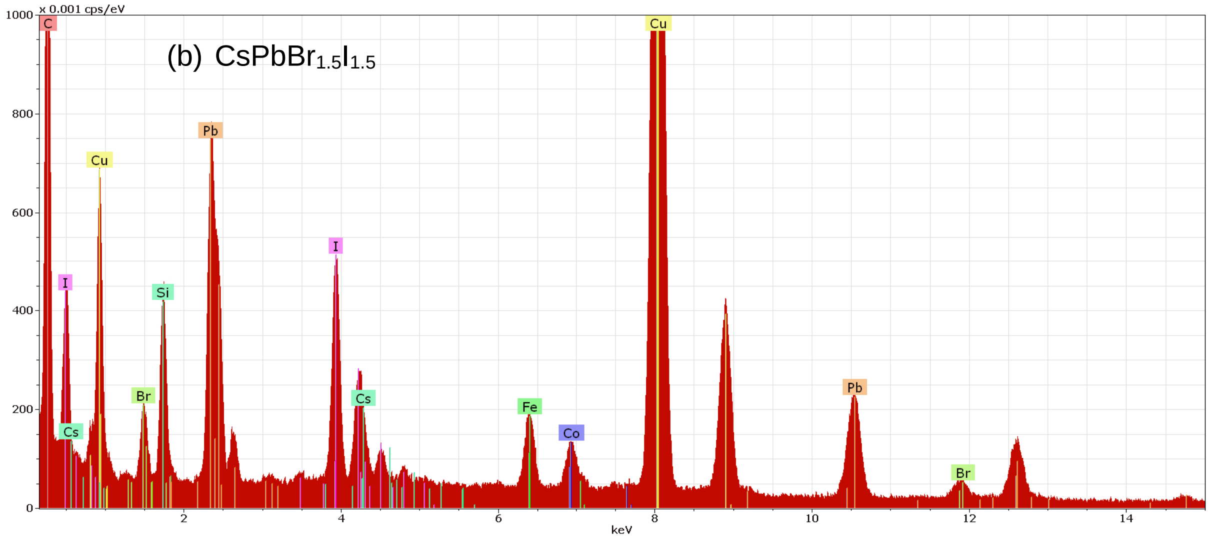

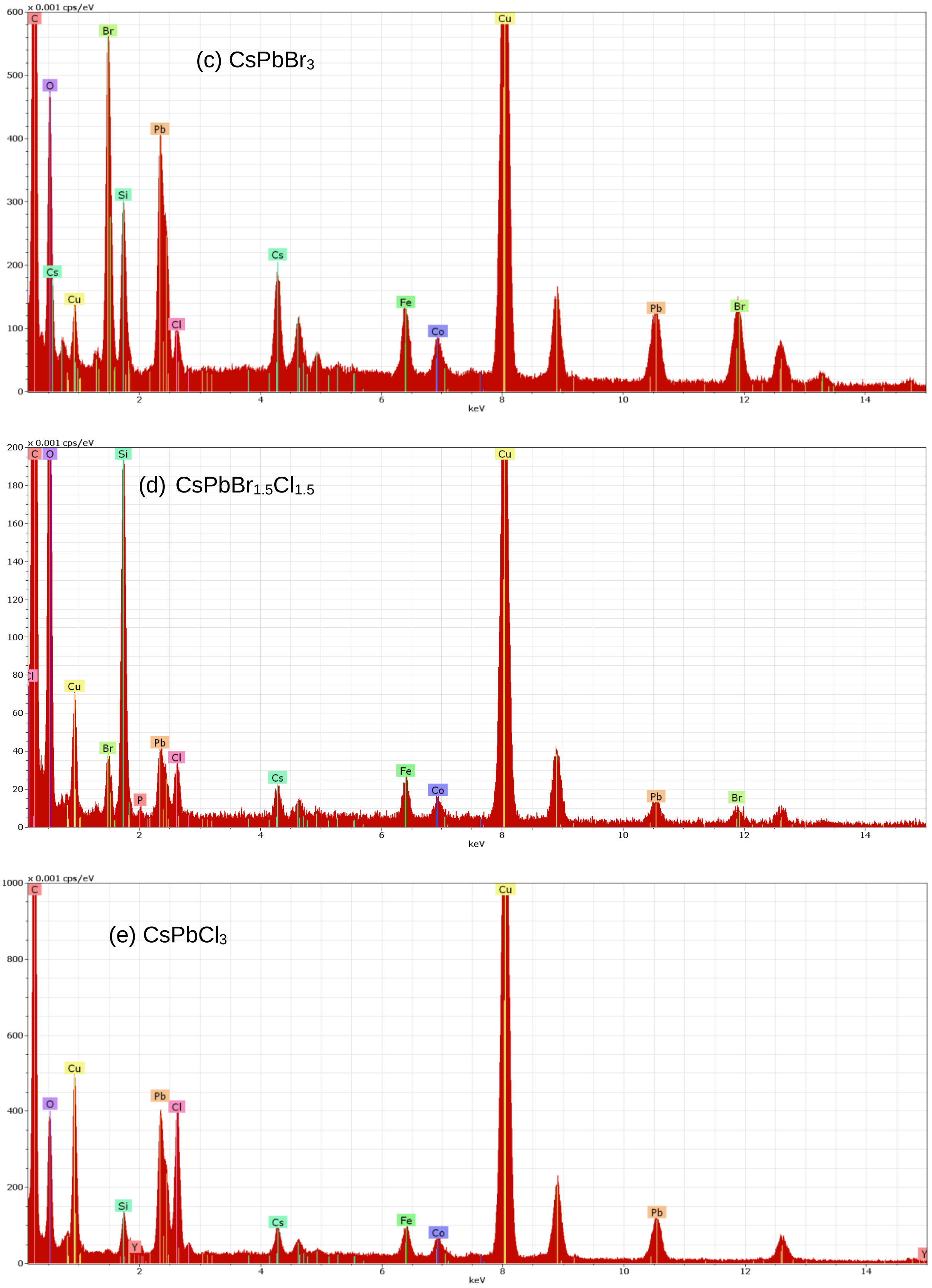

Figure S1. EDS spectra taken during the TEM imaging of (a) $CsPbI_3$, (b) $CsPbBr_{1.5}I_{1.5}$, (c) $CsPbBr_3$, (d) $CsPbBr_{1.5}Cl_{1.5}$ and (e) $CsPbCl_3$.

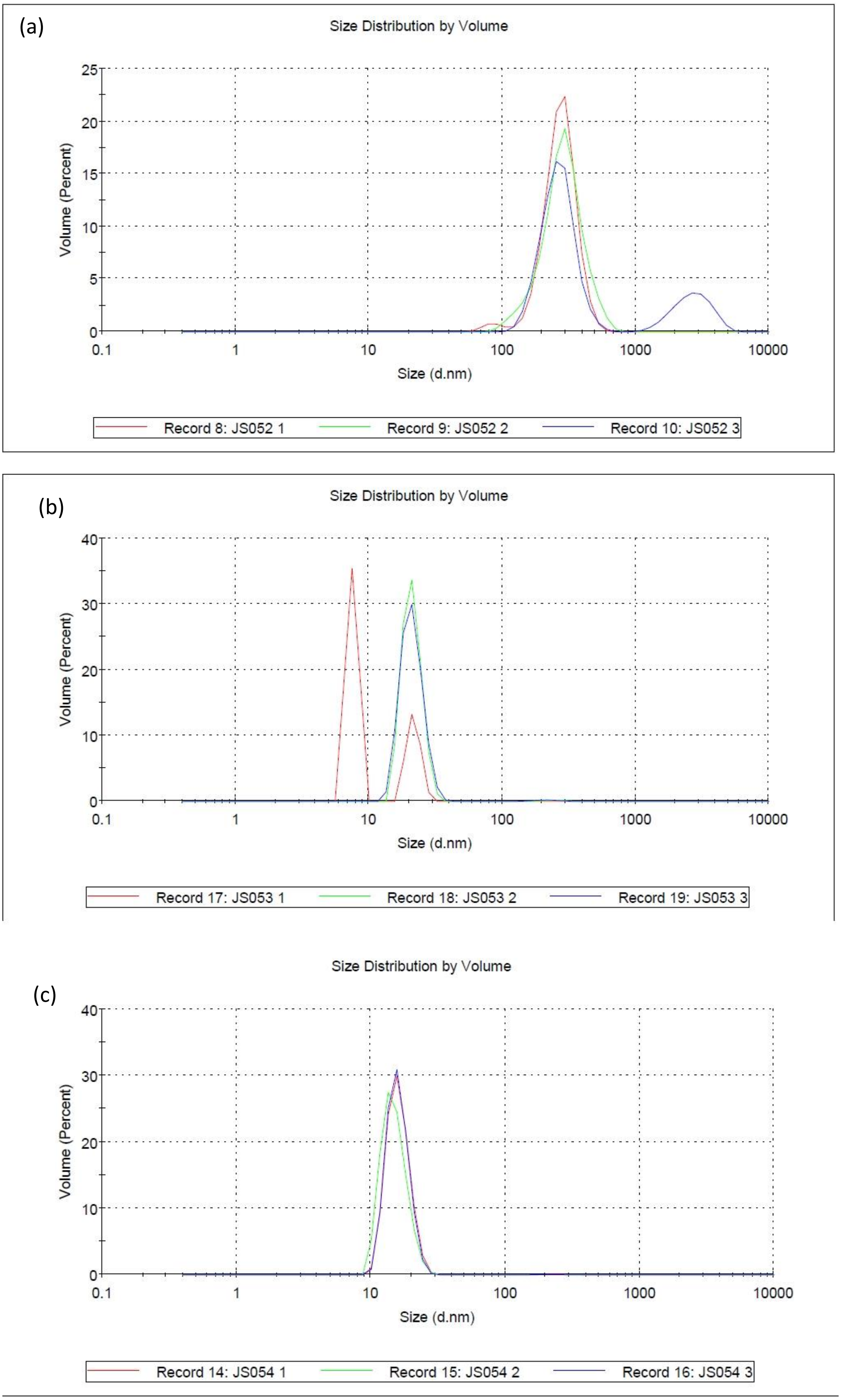
(a)
Size Distribution by Volume
Volume (Percent)
25
20
15
10
5
0
0.1
1
10
100
1000
10000
Size (d.nm)
Record 8: JS052 1
Record 9: JS052 2
Record 10: JS052 3
(b)
Size Distribution by Volume
Volume (Percent)
40
30
20
10
0
0.1
1
10
100
1000
10000
Size (d.nm)
Record 17: JS053 1
Record 18: JS053 2
Record 19: JS053 3
Size Distribution by Volume
(c)
Volume (Percent)
40
30
20
10
0
0.1
1
10
100
1000
10000
Size (d.nm)
Record 14: JS054 1
Record 15: JS054 2
Record 16: JS054 3

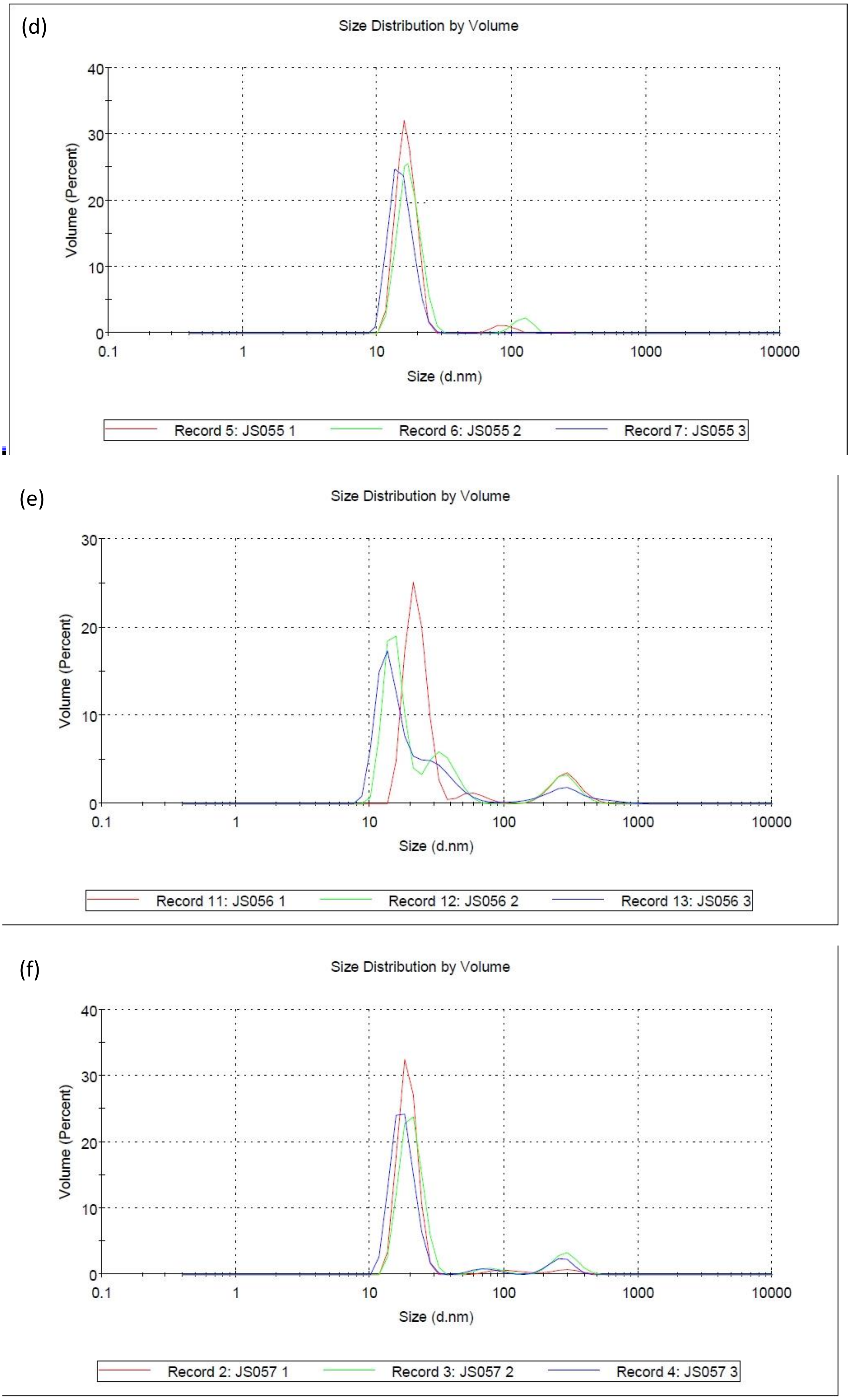

Figure S2. DLS measurements taken for (a) $CsPbBr_2$ sedimented particles (b) $CsPbBr_2$ well dispersed particles, (c) $CsPbBr_{1.5}Cl_{1.5}$, (d) $CsPbBr_{1.5}I_{1.5}$, (e) $CsPbI_3$ and (f) $CsPbCl_3$.

2. Nonlinear optical measurements

In our Z-scan set-up a sample is moved through the focus of the laser beam and the transmitted light is split to be measured with two detectors, one of which has an aperture in front. This allows for collecting simultaneously the open aperture (OA) and closed aperture (CA) Z-scan traces. At each wavelength three consecutive scans are taken: for a 4.66 mm silica glass plate, the solvent and the investigated solution in 1 mm light path glass cuvette. Assuming that n_2 of silica glass is known, this allows one to estimate the beam intensity at the focus, subtract the solvent influence and extrapolate the results to the bulk material. The experimental curves are fitted with the phase shift $\Delta\varphi$, and T parameters using the equations derived by Sheik-Bahae *et al.* [29] . Examples of fitted CA and OA traces are shown in the Figure S1.

Next the following relations are used to calculate the nonlinear refractive index (n_2), nonlinear absorption coefficient (α_2), two-photon absorption cross-section (σ_2) and third-order nonlinear optical susceptibility $\chi^{(3)}$:

$$\Delta\phi_{sample,solution} = \mathrm{X} \cdot \Delta\phi_{measured\ material} + (1 - \mathrm{X})\Delta\phi_{solvent}$$

$$Re\left(\hat{n}_{2,measured\ material}\right) = \frac{\Delta\phi_{measured\ material}}{\Delta\phi_{silica}} \cdot \frac{l_{silica}}{l_{sample}} \cdot n_{2,silica}$$

$$Im\left(\hat{n}_{2,measured\ material}\right) = \frac{1}{4\pi} \cdot \frac{T \cdot \Delta\phi_{measured\ material}}{\Delta\phi_{silica}} \cdot \frac{l_{silica}}{l_{sample}} \cdot n_{2,silica}$$

the nonlinear refractive index:

$$n_2 = Re\left(\hat{n}_{2,measured\ material}\right)$$

nonlinear absorption coefficient:

$$\alpha_2 = \frac{4\pi \cdot Im\left(\hat{n}_{2,measured\ material}\right)}{\lambda}$$

two-photon absorption cross section:

$$\sigma_2 = \frac{\hbar\omega}{N} \cdot \alpha_2, \qquad where:\ N = \frac{N_A \cdot d_{measured\ material}}{M_{measured\ material}}$$

Nonlinear refraction cross section:

$$\sigma_R = \frac{\hbar\omega}{N} \cdot kn_2,$$

where: k is the wavenumber

$\chi^{(3)} -$ cubic (third order) susceptibility Re and Im:

$$Re/Im\left(\chi^{(3)}\right) = \frac{Re/Im(\hat{n}_2) \cdot n^2}{C_1}$$

$$C_1 = 0.039$$

C_1 is conversion factor from cgs to SI

modulus of the complex $\chi^{(3)}$:

$$\left|\chi^{(3)}\right| = \sqrt{Re(\chi^{(3)})^2 + Im(\chi^{(3)})^2}$$

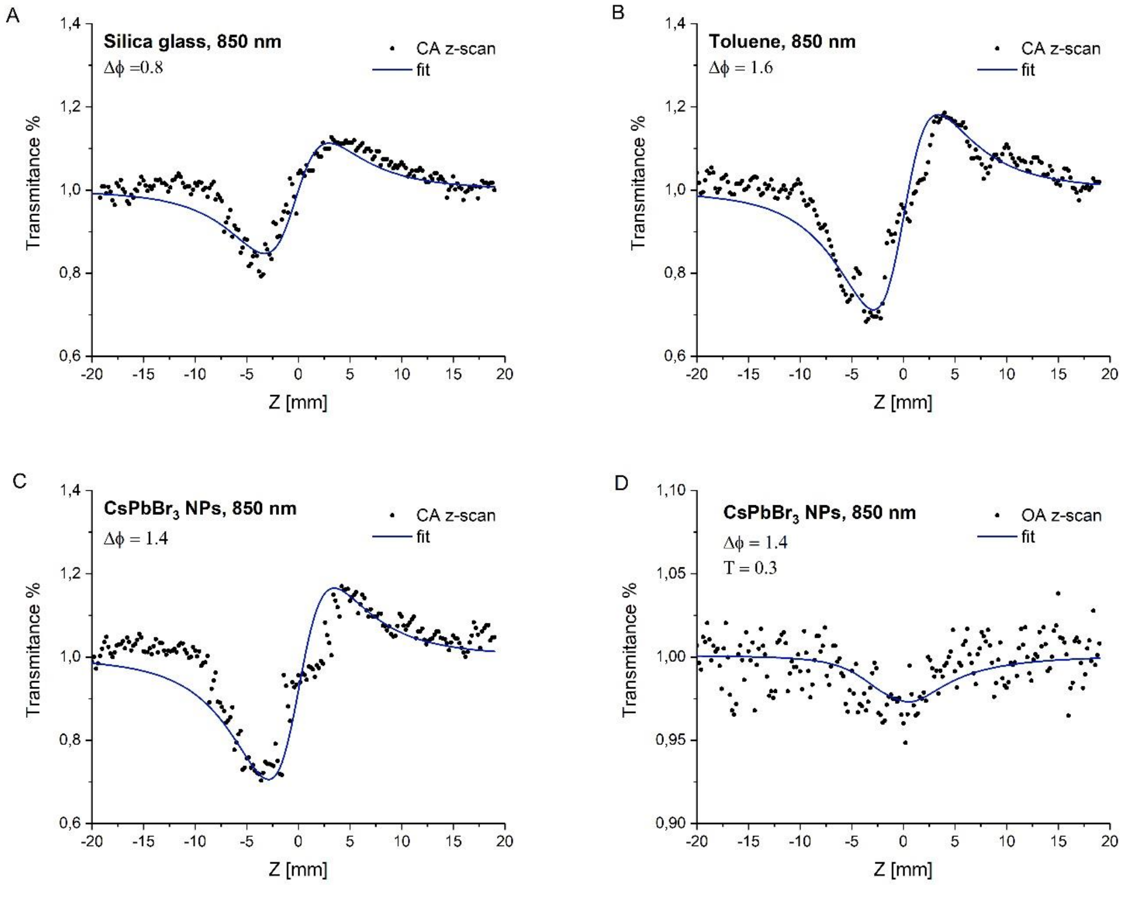

Figure S3. Closed aperture (CA) Z-scan traces with theoretical fits recorded using 850 nm fs laser beam for (a) silica glass, (b) toluene, (c) $CsPbBr_3$ NCs and (d) simultaneously recorded open aperture (OA) Z-scan $CsPbBr_3$ NCs.

In order to determine σ_2 from the emission spectra excited by two photon absorption one has to first acquire the spectra of the reference dyes, with known photoluminescence quantum yield (PLQY) η_{ref} and $\sigma_{2,ref}$ and relate calculated emission intensity to the fluorescence intensity of the measured sample excited in the same conditions. Exemplary set of spectra is shown in Figure S2. Next σ_2 can be calculated using the equation:

$$\sigma_2 = \sigma_{2,ref} \frac{\eta_{ref} \cdot c_{ref}}{\eta \cdot c} \cdot \frac{P_{ref}^2}{P^2} \cdot \frac{I}{I_{ref}} \cdot K$$

where

$$K = \frac{n^2}{n_{ref}^2}$$

is the correction factor for the differences in the refractive index of reference and sample solvents.

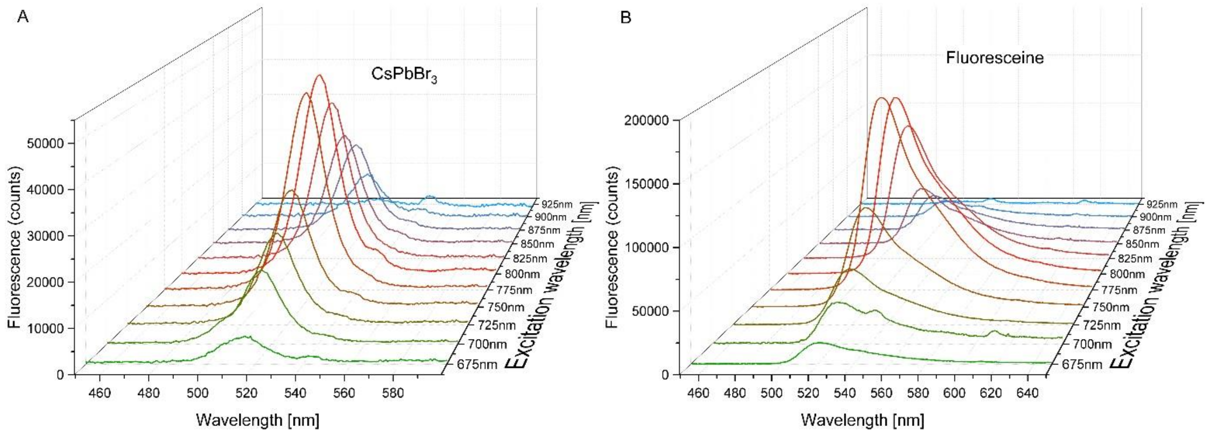

Figure S1. Spectra of the two-photon excited emission of (a) $CsPbBr_3$ NCs and (b) fluorescein excited with the tunable fs laser.
